# Supplementary material for: A cross-cultural examination of temporal orientation through everyday language on social media
Source: PLoS One. 2024 Mar 8;19(3):e0292963. doi: 10.1371/journal.pone.0292963 (PMC10923455; doi:10.1371/journal.pone.0292963)
Supplement: S1 Table — (DOCX) [file pone.0292963.s001.docx]

**Supporting Information**

**S1 Table. Previous Sampling Examples.**

| **Article** | **Sample Size** | **Sample Demographics** | **Measurement** |
| --- | --- | --- | --- |
| Guo et al. (2012) | N1a_EuroCan = 98  N1a_CHN = 115;  N1b_EuroCan = 99  N1b_CHN = 88;  N2_EuroCan = 88  N2_CHN = 39;  N3_EuroCan = 171  N3_CHN = 189 | All studies recruited  undergraduate students. | Experiments;  Self-report questionnaires |
| Ji et al. (2009) | N1_EuroCan = 160  N1_CHN = 183;  N2_EuroCan = 83  N2_CHN = 57;  N3_EuroCan = 34  N3_CHN = 41;  N4_EuroCan = 59  N4_CHN = 67  N5_EuroCan = 49  N5_CHN = 57 | All studies recruited undergraduate students. | Experiments;  Self-report questionnaires |
| Sircova et al. (2014) | N_US = 565  N_CHN1 = 356  N_CHN2 = 924 | US sample was archived data from the International Research Network;  Chinese samples were drawn from local organizations. | Self-report questionnaires (ZIPI) |
| Ji et al. (2001) | N1_US = 56  N1_CHN = 63;  N2_US = 49  N2_CHN = 63;  N3_US = 41  N3_CHN = 58;  N4_US = 140  N4_CHN = 181 | All studies recruited undergraduate students. |  |
| Wang et al. (2011) | N1_US = 99  N_CHN = 208 | Undergraduate students |  |
