# Supplementary material for: A cross-cultural examination of temporal orientation through everyday language on social media
Source: PLoS One. 2024 Mar 8;19(3):e0292963. doi: 10.1371/journal.pone.0292963 (PMC10923455; doi:10.1371/journal.pone.0292963)
Supplement: Attachment — Submitted filename: Comments PONE-D-23-03984_reviewer(2).docx [file pone.0292963.s003.docx]

The manuscript discusses an interesting issue of global importance; A Cross-cultural Examination of Temporal Orientation Through Everyday Language on Social Media, while the manuscript is well delivered. I provide some comments for improvement.

The sentence ‘Implications of the findings are discussed’ should remove from the abstract.

Statement of the research problems need a more clear articulation.

Can you please provide the equation of the logistic regression classification model and Randomized Principle Component Aanalysis with empirical model?

Please add a bit more the main findings.
