# Supplementary material for: A cross-cultural examination of temporal orientation through everyday language on social media
Source: PLoS One. 2024 Mar 8;19(3):e0292963. doi: 10.1371/journal.pone.0292963 (PMC10923455; doi:10.1371/journal.pone.0292963)
Supplement: Attachment — Submitted filename: Response to Reviewers.docx [file pone.0292963.s004.docx]

Dear Dr. Chen,

We appreciate the opportunity to revise the paper and are very grateful for the helpful feedback you and the reviewers provided. We have made every effort to address the concerns raised and incorporate helpful suggestions in our paper. As a result, we believe that the paper is considerably better due to the constructive feedback. In the followings, we provide detailed responses to all the reviewer concerns and believe we have addressed them.

**Abstract**

Reviewer 1 has suggested to remove the sentence “Implications of the findings are discussed’ should be removed from the abstract”.

- We have eliminated this sentence from the abstract.

Reviewer 2 has suggested spelling out “United States of America” in full for the first time in the abstract.

- We have spelled it out in full.

Reviewer 2 has suggested that it is essential to indicate the implications of the findings in the abstract and offer a policy recommendation.

- Please see our revision at the bottom of the Abstract section on page 2.

**Introduction**

Review 1 has suggested that statement of the research problems needs a clearer articulation.

- Please see the last paragraph of Introduction/bottom paragraph on page 5. We added a sentence to clearly define the research problem and objective.

**Method**

Both reviewers 1 and 2 have asked to “provide the equation and output of the logistic regression classification model and Randomized Principal Component Analysis with empirical model”

- We added the equation in the last paragraph of the Method section on page 9 and additional examples of other studies that have followed similar methodology approaches in the past.

Review 2 has suggested that under the method section, it is important for the authors to state the research design, whether it is a mixed method or a qualitative study.

- We have clarified the language in the beginning of Method section to be “mixed method”.

**Discussion**

Review 1 has suggested to add a bit more the main findings.

- We have re-iterated the main findings at the start of the Discussion section.

Reviewer 2 has suggested that previous studies have generally posited temporal differences in past and future between Eastern and Western cultures. The authors need to reference these previous studies.

- We have referenced these previous studies in the manuscript.

**Conclusion**

Reviewer 2 has suggested a need to highlight the implications of the findings for culture and social media influence.

- Please see the revision in the last paragraph of the Discussion section/on the bottom paragraph of page 15.

Reviewer 2 has suggested a need to present a conceptual or theoretical framework to guide the study.

- We appreciate this feedback. As this paper is an empirical test of the temporal orientation using a new methodology (i.e., social media language), there is no strong conceptual or theoretical framework. Rather, our paper is driven by the phenomenology of temporal orientation. As mentioned in the abstract, this is “an inductive approach” rather than a top-down theoretical deductive approach. We have also emphasized this in our introduction and in the discussion too. Finally, in our discussion, we have pointed out how this study contributes to the current trichotomous framework of temporal orientation and call for an expanded view.

We again thank you and the reviewers for the helpful feedback. In the above, we provide detailed responses to all the reviewer concerns and believe we have addressed them. However, if you should feel that additional issues are needed, we would be happy to accommodate or provide more information.

Sincerely,

Daphne Hou
