# Supplementary material for: A cross-cultural examination of temporal orientation through everyday language on social media
Source: PLoS One. 2024 Mar 8;19(3):e0292963. doi: 10.1371/journal.pone.0292963 (PMC10923455; doi:10.1371/journal.pone.0292963)
Supplement: Attachment — Submitted filename: PONE-D-23-03984_R1_reviewer-1.docx [file pone.0292963.s005.docx]

Authors have made great effort to the betterment of the manuscript. It has desire to the public and the literature in terms of new communication technology and methodological approach. I recommend for getting it published if it fit the journal scope.
