# Supplementary material for: A cross-cultural examination of temporal orientation through everyday language on social media
Source: PLoS One. 2024 Mar 8;19(3):e0292963. doi: 10.1371/journal.pone.0292963 (PMC10923455; doi:10.1371/journal.pone.0292963)
Supplement: Attachment — Submitted filename: Reviewer comments.docx [file pone.0292963.s002.docx]

**Abstract**

There is the need to mention United States of America in full for the first time in the abstract.

It is important to indicate the implications of the findings in the abstract and offer a policy recommendation.

**Method**

Under the method section, it is important for the authors to state the research design whether it is a mixed method or a qualitative study.

….We used these as input features in a logistic regression classification model along with a feature selection pipeline which uses a univariate feature selection (with a family wise error rate of 60) and Randomized Principle Component Aanalysis to reduce the dimensions and predict dummy variables representing whether each message was annotated as past, present, and future. The authors presented this statement in their methods but there is not regression output in the results.

**Discussion**

First, previous studies have generally posited temporal differences in past and future between Eastern and Western cultures. The authors need to reference these previous studies.

**Conclusion**

There is the need for the authors to highlight the implications of the findings for culture and social media influence.

There is the need for the authors to present a conceptual or theoretical framework to guide the study.
